# Association of anti-β2-glycoprotein I/HLA-DR complex antibody with arterial thrombosis in female patients with systemic rheumatic diseases

**DOI:** 10.1186/s13075-023-03175-8

**Published:** 2023-10-06

**Authors:** Katsuhiko Yoneda, Yo Ueda, Kenji Tanimura, Hisashi Arase, Hideto Yamada, Jun Saegusa

**Affiliations:** 1 Department of Rheumatology and Clinical Immunology, Kobe University Graduate School of Medicine, 7-5-1 Kusunoki-Cho, Chuo-Ku, Kobe, 650-0017 Japan; 2 Department of Obstetrics and Gynecology, Kobe University Graduate School of Medicine, Kobe, Japan; 3 Department of Immunochemistry, Research Institute for Microbial Diseases, Osaka University, Suita, Osaka 565-0871 Japan; 4 Laboratory of Immunochemistry, World Premier International Immunology Frontier Research Centre, Osaka University, Suita, Osaka 565-0871 Japan; 5 Center for Recurrent Pregnancy Loss, Teine Keijinkai Hospital, Sapporo, Japan

**Keywords:** Antiphospholipid autoantibody, Antiphospholipid syndrome, Cardiovascular diseases, Thrombosis, Rheumatic disease

## Abstract

**Background:**

β2-glycoprotein I (β2GPI) complexed with human leukocyte antigen DR (β2GPI/HLA-DR) was found to be a major autoantibody target in antiphospholipid syndrome (APS). This study aimed to reveal the association between anti-β2GPI/HLA-DR antibodies and vascular thromboses in women with systemic rheumatic diseases.

**Methods:**

We conducted a retrospective longitudinal study. We measured anti-β2GPI/HLA-DR antibodies and compared them with anti-phospholipid antibody (aPL) profiles and the adjusted global antiphospholipid syndrome score (aGAPSS). Using receiver operating characteristic (ROC) analysis, we determined the best cut-off value for arterial thrombosis. We also evaluated the validity of anti-β2GPI/HLA-DR antibodies by adding to conventional cardiovascular risk factors in multivariate logistic analysis.

**Results:**

We evaluated 704 patients, including 66 (obstetric or thrombotic) APS, 13 primary APS, and 78 asymptomatic aPL carriers. Seventy-seven patients had a history of arterial thrombosis, and 14 patients had both arterial and venous thrombosis. These 14 patients, as well as patients with aGAPSS > 10 or triple-positive aPL profiles, displayed high anti-β2GPI/HLA-DR antibody titers. The ROC curve showed a sensitivity, specificity, and area under the curve (AUC) for arterial thrombosis of 33.8%, 91.4%, and 0.6009, respectively, with a cut-off value of 172.359 U/mL. The anti-β2GPI/HLA-DR antibody positivity using this cut-off value yielded an odds ratio of 5.13 (95%CI: 2.85–9.24), significantly improving the AUC from 0.677 to 0.730.

**Conclusion:**

Anti-β2GPI/HLA-DR antibodies are associated with arterial thrombosis in female patients with systemic rheumatic diseases.

**Supplementary Information:**

The online version contains supplementary material available at 10.1186/s13075-023-03175-8.

## Introduction

Antiphospholipid syndrome (APS) is an autoimmune disorder characterized by thrombotic or obstetric events with persistent aPL antibodies [1]. β2glycoprotein I (β2GPI) is one of the primary phospholipid-binding proteins recognized by aPL [2]. When the circular type β2GPI binds to negatively charged molecules, such as an immobilized anionic phospholipid like cardiolipin (CL) or negatively charged solid plates [3], it transforms into a linear domain that contains a pathogenic epitope [4]. The binding of β2GPI to phospholipids is closely linked to the risk of thrombosis in APS [5].

We currently apply anti-β2GPI (aβ2GPI) and anti-CL (aCL) antibody assays to detect aPL antibodies, which are pretty good at picking out APS patients. However, standardized quantitative solid-phase enzyme-linked immunosorbent assay (ELISA) cannot fully diagnose all APS patients with clinical manifestations satisfying the criteria [6].

We have recently discovered that β2GPI could also bind to specific major histocompatibility complex (MHC) class II molecules and serve as one of the main autoantibody targets in the development of APS [7]. Anti-β2GPI/HLA-DR antibodies can specifically detect patients with recurrent pregnancy loss [8] or with idiopathic chronic limb ulcers [9] and are believed to provide two advantages with respect to the pathogenesis of APS. First, anti-β2GPI/HLA-DR antibodies have an enhanced ability to bind to disease-susceptible HLA alleles [7]. Second, β2GPI/HLA-DR molecules can appear on the endothelial cell surface under inflammatory conditions, such as in elevated levels of interferon (IFN)-gamma (IFNγ) and tumor necrosis factor-alpha (TNFα) [7, 10, 11]. These mechanisms may explain the positive correlation of increased IFN type I expression with anti-β2GPI antibodies [12] and justify the finding that aPL-positive individuals with systemic rheumatic diseases are at a greater risk of thrombosis than those without systemic rheumatic diseases [13].

Therefore, we hypothesized that anti-β2GPI/HLA-DR antibodies could be highly expressed in patients with systemic rheumatic diseases, possibly contributing to thrombosis.

Patients suffering from systemic rheumatic diseases (in particular with disorders characterized by systemic inflammation) are thought to be at increased risk for developing cardiovascular events [14]. Hence, this study aimed to determine the association between anti-β2GPI/HLA-DR antibodies and vascular thrombosis in women with systemic rheumatic diseases and to elucidate whether the anti-β2GPI/HLA-DR antibodies represent an additional risk factor.

## Patients and methods

### Patients and ethics

This was a single-centered, cross-sectional study. Our study followed the Declaration of Helsinki guidelines and was approved by the Institutional Review Boards of our centers (approval no. B190102 at Kobe University Hospital); the project “Baby and Infant in Research of health and Development to Adolescent and Young adult” received support from the Japan Agency for Medical Research and Development (AMED). This project focused on women aged above the reproductive age, who were planning a pregnancy or those with a pregnancy history.

All included participants were female patients who followed up in our department from April 2020 to December 2021 with provided written informed consent.

We assessed medical records, questionnaires, and laboratory tests. We retrieved information regarding thrombotic events, conventional cardiovascular risk factors, and medications of corticosteroids, hydroxychloroquine, immunosuppressive agents, and oral anticoagulants.

### Classification criteria for systemic rheumatic diseases

Patients with APS fulfilled the updated Sapporo–Sydney classification criteria [1], while asymptomatic aPL carriers only fulfilled the laboratory criteria. Patients with systemic lupus erythematosus (SLE) met the updated 1997 American College of Rheumatology (ACR) classification criteria for SLE or the 2012 Systemic Lupus International Collaborating Clinics classification criteria [15, 16]. Patients with mixed connective tissue disease (MCTD) were classified using the 2019 Diagnostic criteria for MCTD [17], but those who met the diagnosis of SLE were classified as SLE. Patients with systemic sclerosis (SSc) fulfilled the 2013 ACR/European Alliance of Associations for Rheumatology (ACR/EULAR) classification criteria [18]. Patients with Sjögren’s syndrome (SjS) satisfied the ACR/EULAR 2016 classification criteria [19]. Some patients with rheumatic autoimmune diseases who fulfilled the classification criteria were diagnosed with idiopathic inflammatory myositis (IIMs), Bechet’s disease, ANCA-associated arthritis, and large-vessel arteritis, including Takayasu’s arteritis and giant cell arteritis. The remaining patients were evaluated based on clinical diagnoses by a rheumatologist. Two women who did not fulfill the classification criteria for rheumatic autoimmune disorders were included as unclassified connective tissue disease (UCTD).

### Data collection

#### Demographic and study characteristics

Our survey considered factors such as age, disease duration, body mass index (BMI), current or past smoking, pack-year smoking index, arterial hypertension (≥ 140/90 mmHg or using antihypertensive drugs), dyslipidemia (total serum cholesterol or triglyceride levels > 230 mg/dL and 150 mg/dL, respectively, or being on lipid-lowering medications, such as statins), diabetes (plasma glycated hemoglobin levels ≥ 6.5% and fasting plasma glucose 126 mg/dL, 2 h after oral glucose tolerance test or casual plasma glucose above 200 mg/dL, respectively, or using insulin or oral antidiabetic drugs), past or present family history of stroke or myocardial infarction, and personal history of thrombosis.

#### Vascular thrombotic morbidity

Thrombotic complications were defined as previously described [20]. Arterial thrombotic events were confirmed by their clinical features or imaging studies using computed tomography (CT) scanning, magnetic resonance imaging (MRI), or angiography (arterial thromboses are compiled in Figure S1B). A transient ischemic attack (TIA) was excluded due to the absence of modalities.

Venous thrombotic events consisted mostly of venous thromboembolism, which were detected not only by their clinical features but also by imaging studies using computed tomography, angiography, or scintigraphy. Seven patients with retinal venous thrombosis (retinal vein occlusion) were diagnosed appropriately by an ophthalmologist. A patient with atrial thrombosis was detected by transthoracic echocardiography. Thus, the study classified these events as venous thrombotic events.

Patients without these thrombotic events were considered to have no thrombosis.

#### Obstetric morbidity

Pregnancy complications were defined as follows:

- Three or more recurrent miscarriages at gestational age < 10 weeks
- Fetal death at gestational age > 10 weeks
- Premature birth before the 34th week of gestation due to hypertensive disorders of pregnancy, such as preeclampsia or placental insufficiency.

#### Conventional cardiovascular risk factors for arterial thrombosis and adjusted global APS score calculation

The assessment of cardiovascular risk, age, arterial hypertension, diabetes, smoking habit, dyslipidemia, and BMI was considered to be based on some traditional risk factors [21]. The adjusted global APS score (aGAPSS) [22] was calculated as previously described. aGAPSS is a composite measure to estimate the risk of arterial thrombosis; aGAPSS > 10 points indicates a significant risk for ischemic stroke [23] and cardiovascular disease [24]. aGAPSS ≥ 14 (aGAPSS > 13) points are associated with the highest risk of clinical recurrence in patients with APS [25]. Referring to a previous study for risk stratification with aGAPSS [26], the aGAPSS cluster was categorized as follows: none (< 1 point), very low (1–3 points), low (4–5 points), middle (6–9 points), high (10–13 points), and very high (≥ 14 points).

#### Detection of autoantibodies

Serum samples were collected from 704 patients. Of these, 343 patients were tested for the aPL panel using commercially available chemiluminescent immunoassay (CIA) kits, including both aCL and anti-β2GPI IgG/IgM antibodies, and evaluated multiple aPL antibodies. Positive aCL was defined as aCL IgG/IgM levels > 20 U/mL; positive anti-β2GPI was defined as anti-β2GPI IgG/IgM levels > 20 U/mL. This study adopted the aPL panel test, QUANTA Flash® (INOVA Diagnostics), detected by HemosIL ACL AcuStar® (Instrumentation Laboratory (IL)), which was provided by IL Japan (Tokyo, Japan). Their cut-off values were assigned to 20 U/mL for positivity of anti-CL or anti-β2GPI IgG/IgM, reported as the 99th percentile of the distribution of 250 healthy donors [27]. However, these cut-off values were refined to lower than 20 U/mL, using 626 healthy donors [28]. These revised cut-off values yielded a slight decrease in specificity. Hence, we have adopted the cut-off values to define positivity as 20 units/mL to compare with anti-β2GP1/HLA-DR antibodies.

Additionally, we retrospectively surveyed the following aPL autoantibodies detected by commercially available enzyme immunoassay (EIA) solid-phase kits: aCL IgG antibodies and aCL β2 glycoprotein I (anti-CLβ2GPI) IgG antibodies. Positive aCL was defined as aCL IgG levels > 10 U/mL (10.2 U/mL is 99th percentile value); positive anti-CLβ2GPI was defined as anti-CLβ2GPI IgG levels > 3.5 U/mL (1.8 U/mL is 99th percentile value), as recommended by the manufacturer of each kit.

Lupus anticoagulant (LA) was measured according to the guidelines [29]. For positive LA using silica clotting time, the normalized screen ratio was defined as ≥ 1.20.

Positivity of either 1, 2, or 3 assays, including aCL IgG/IgM, anti-β2GPI IgG/IgM antibodies using CIA or EIA kits, and LA, was defined as single, double, and triple-positive, respectively.

Non-criteria aPL autoantibodies, such as anti-phosphatidylserine-prothrombin (aPS/PT), were not measured since these are not yet covered by medical insurance.

#### Measurement of levels of antibodies for β2GPI/HLA-DR

Anti-β2GPI/HLA-DR antibodies (Revorf Co., Ltd. Tokyo, Japan.) were quantified based on a previously reported method [8]. The reference value was determined as 73.3 U/mL with a 99th percentile value in 374 healthy control participants after outlier removal [30].

### Statistical analyses

All statistical analyses were performed using R (version 4.1.2).

The data are expressed in positive or negative numbers, percentages for categorical variables, mean ± standard deviation, or median and interquartile range. The chi-squared and Fisher’s exact tests were used for categorical variables, as appropriate. The Mann–Whitney *U* and Kruskal–Wallis tests, with post hoc comparisons using the Steel–Dwass tests, were used to compare continuous variables between groups. A two-tailed *p*-value < 0.05 indicated a significant difference between arterial and non-arterial thrombosis groups.

Receiver operating characteristic (ROC) analyses were conducted to demonstrate the optimal cut-off value of anti-β2GPI/HLA-DR antibodies in differentiating arterial thrombosis from non-arterial thrombosis. We evaluated the performances of different aPL antibodies by calculating the area under the curve (AUC), sensitivity, and specificity of the suggested cut-off values by using Fisher’s exact tests and the Youden index. Spearman’s Rank correlation coefficient was calculated to analyze the correlation between anti-β2GPI/HLA-DR antibody titers and aβ2GPI IgG antibody or aCL IgG antibody titers.

Multivariate logistic regression models were prepared to estimate the risk of arterial thrombosis associated with potential predictors, including clinical or sociodemographic variables such as age, disease duration, BMI, pack-year smoking index, current doses of prednisolone, dyslipidemia, arterial hypertension, diabetes, and the cut-off of anti-β2GPI/HLA-DR antibodies. Variables in these models were based on conventional cardiovascular risk factors for arterial thrombosis, adjusted global APS score, and significant factors in univariate analysis. The variance inflation factor (VIF) was used to check multicollinearity. The AUC was evaluated and compared the discrimination efficacy of the multivariate model. Further, the Delong test for comparison of AUCs, Net reclassification improvement (NRI), and integrated discrimination improvement (IDI) analyses were conducted to evaluate and compare potential net benefits by adding the threshold of anti-β2GPI/HLA-DR antibody. Calibration plots were calculated with the regression modeling strategies (rms) package in R. The outcome of the odds ratios is presented together with the 95% confidence interval (CI). The level of statistical significance was set at a two-tailed α-value of 0.05 by default.

### Multiple imputation

BMI, disease duration, and pack-year smoking had missing values. We adjusted the dataset with multiple imputation (MI) by chained equation (MICE) package (version 3.15.0) [31]. We replaced missing values with complementary values by using the following covariates to produce 20 filled-in datasets through the imputation process: age, disease duration, height, body weight, BMI, smoking history, pack-year smoking index, dyslipidemia, arterial hypertension, diabetes, anti-β2GPI/HLA-DR antibodies, aGAPSS, with or without arterial thrombosis, several laboratory data (platelet cell count, C-reactive protein, erythrocyte sedimentation rate, IgG subtype, complements component 3 and 4, different aPL titers), presence or absence of current medications, which include current doses of prednisolone, hydroxychloroquine, or immunosuppressive agents.

## Results

### Demographic and clinical characteristics of enrolled patients

Among the 721 patients, 704 samples were collected in the dataset, and 17 were excluded owing to lost visits (Figure S1A). We identified 77 patients with histories of arterial thrombosis, including only arterial thrombosis (*n* = 63) and both arterial and venous thrombosis (*n* = 14). The group of non-arterial thrombosis (*n* = 627) included patients with no history of thrombosis (*n* = 583) or with venous thrombosis only (*n* = 44). Among the 77 patients with arterial thrombosis, the most common manifestation was cerebral infarction (Figure S1B).

Clinical and demographic characteristics stratified according to the presence of arterial thrombosis are reported in Table 1. Some cardiovascular risks, such as age, arterial hypertension, smoking, and current dosage of prednisolone, were detected more frequently in patients with arterial thrombosis than among participants with non-arterial thrombosis (Table 1). Distributions of aPL autoantibodies, particularly aβ2GPI IgG subtypes, LA positivity, anti-β2GPI/HLA-DR antibody levels, and aGAPSS, tended to be higher in the arterial thrombosis group than in the non-arterial thrombosis group.

**Table 1:** Baseline characteristics of patient epidemiology and laboratory findings

Variables	Subjects		Thrombosis		*p*-value^2^
		Overall	Non-arterial	Arterial	
		all = 704^1^	*N* = 627^1^	*N* = 77^1^	
Demographics					
Age, mean (± SD), years	704	57.1 ± 15.8	56.7 ± 15.9	60.5 ± 14.4	0.046
Female sex, *n* (%)		704 (100%)	627 (100%)	77 (100%)	1.000
Disease duration, median (IQR), years	699	12 (6, 21)	12 (6, 20)	14 (5, 23)	0.405
The maximum dose of prednisolone, median (IQR), mg/day	622	25 (5, 45)	20 (3.75, 45)	30 (10, 50)	0.020
Current dose of prednisolone, median (IQR), mg/day	704	2 (0, 5)	2 (0, 5)	4.5 (1, 5.5)	< 0.001
Conventional cardiovascular risk factors					
BMI, mean (± SD), kg/m^2^	458	21.5 ± 4.0	21.4 ± 4.1	22.0 ± 3.4	0.124
Smoking history, *n* (%)					
No		528 (75%)	475 (76%)	53 (69%)	0.133
Former		84 (12%)	70 (11%)	14 (18%)	
Current		14 (2.0%)	11 (1.8%)	3 (3.9%)	
Unknown		78 (11%)	71 (11%)	7 (9.1%)	
Pack-year smoking, mean (± SD)	620	2.20 ± 6.68	1.90 ± 6.01	4.66 ± 10.4	0.026
Arterial hypertension, *n* (%)		169 (24%)	137 (22%)	32 (42%)	< 0.001
Diabetes mellitus,* n* (%)		76 (11%)	64 (10%)	12 (16%)	0.151
Dyslipidemia,* n* (%)		203 (29%)	179 (29%)	24 (31%)	0.632
Family History of thrombosis, *n* (%)					
Coronary diseases		16 (2.3%)	16 (2.5%)	0 (0%)	1.000
Stroke		34 (4.8%)	31 (4.9%)	3 (3.9%)	1.000
Autoantibodies for aPL					
aCL IgG [EIA], median (IQR), U/mL	304	6.00 (3.00, 10.25)	6.00 (2.70, 9.00)	7.99 (3.00, 21.50)	0.060
aCLβ2GPI IgG [EIA], median (IQR), U/mL	378	0.69 (0.69, 1.19)	0.69 (0.69, 1.19)	0.70 (0.69, 1.99)	0.022
aCL IgG [CLIA], median (IQR), U/mL	343	5.45 (3.73, 9.20)	5.35 (3.68, 8.75)	6.95 (4.00, 14.50)	0.050
aCL IgM [CLIA], median (IQR), U/mL	343	2.20 (1.30, 4.40)	2.10 (1.30, 4.35)	2.50 (1.60, 4.85)	0.339
aβ2GPI IgG [CLIA], median (IQR), U/mL	343	6.39 (6.39, 10.05)	6.39 (6.39, 8.90)	6.39 (6.39, 42.95)	0.006
aβ2GPI IgM [CLIA], median (IQR), U/mL	343	1.09 (1.09, 1.85)	1.09 (1.09, 1.80)	1.09 (1.09, 1.88)	0.802
LA test, Positive no./total no	368	83/368 (23%)	59/314 (19%)	24/54 (44%)	< 0.001
Anti-β2GPI/HLA-DR, median (IQR), U/mL	704	34.15 (11.92, 80.21)	33.43 (11.82, 74.81)	61.61 (13.36, 256.39)	0.004
APS profile and adjusted global APS score (aGAPSS)					
Patients of APS / aPL carrier, n(%)					
Fulfill the APS classification criteria		66 (9.4%)	32 (5.1%)	34 (44%)	< 0.001
Primary APS (PAPS)		13 (1.8%)	9 (1.4%)	4 (5%)	< 0.001
Secondary APS (SAPS)		53 (7.5%)	23 (3.6%)	30 (39%)	< 0.001
Catastrophic APS (CAPS)		0 (0%)	0 (0%)	0 (0%)	1.000
Asymptomatic aPL carrier		78 (11%)	78 (12%)	0 (0%)	1.000
aGAPSS, median (IQR)	368	3.00 (0.00, 5.00)	1.00 (0.00, 4.00)	4.00 (1.50, 12.25)	< 0.001
Other laboratory data					
PLT, median (IQR), × 10^4^/μL	702	23 (19, 27)	23 (19, 28)	23 (19, 26)	0.427
CRP, median (IQR), mg/dl	702	0.04 (0.02, 0.15)	0.04 (0.02, 0.14)	0.05 (0.02, 0.19)	0.409
IgG, median (IQR), mg/dl	527	1,230 (986, 1,531)	1,234 (993, 1,535)	1,187 (942, 1,512)	0.436
IgA, median (IQR), mg/dl	213	235 (164, 336)	236 (167, 333)	232 (149, 380)	0.998
IgM, median (IQR), mg/dl	211	83 (51, 119)	85 (52, 119)	72 (44, 117)	0.462
C3, median (IQR), mg/dl	316	84 (71, 100)	83 (70, 98)	92 (74, 105)	0.065
C4, median (IQR), mg/dl	311	17 (12, 23)	17 (12, 23)	15 (12, 22)	0.782
Anti-DNA [RIA], median (IQR), IU/mL	151	5.00 (2.00, 11.00)	5.00 (2.00, 11.50)	4.50 (1.99, 8.25)	0.530
Anti-dsDNA IgG [EIA], median (IQR), IU/mL	123	2.40 (1.15, 6.10)	2.30 (1.05, 6.10)	3.45 (2.10, 6.08)	0.294
Anti-U1RNP antibody, Positive no. /total no	404	153/404 (38%)	136/355 (38%)	17/49 (35%)	0.754
Anti-Sm antibody, Positive no. /total no	368	88/368 (24%)	79/322 (25%)	9/46 (20%)	0.580
Anti-SS-A/Ro antibody, Positive no. /total no	485	213/485 (44%)	186/429 (43%)	27/56 (48%)	0.567
RF, median (IQR), IU/mL	400	34 [14, 102]	35 [14, 101]	20 [11, 99]	0.283

*Abbreviations: APS*, antiphospholipid antibody syndrome; *aGAPSS*, adjusted Global Antiphospholipid Syndrome Score; *aCL*, anticardiolipin antibody; *aβ2GPI*, anti-β2GPI antibody; *BMI*, body mass index; *CIA*, chemiluminescent immunoassay; *CRP*, C-reactive protein; *EIA*, enzyme immunoassay; *IQR*, interquartile range; *LA*, lupus anticoagulant; *PLT*, platelet; RF, rheumatoid factor; *SD*, standard deviation; *Sm*, Smith (antigen); *SS-A/Ro*, Sjögren syndrome A/Ro (antigen); *U1RNP*, U1 small nuclear ribonucleoprotein (antigen)

^1^*n* (%) for categorical data; mean ± SD or median (IQR) for qualitative data

^2^Pearson chi-square test (or chi-square test with the Yates continuity, or Fisher’s exact test if appropriate) for categorical data, and the Mann–Whitney *U* test for qualitative data

### Titers of anti-β2GPI/HLA-DR antibodies in different clinical groups

**Fig. 1:**
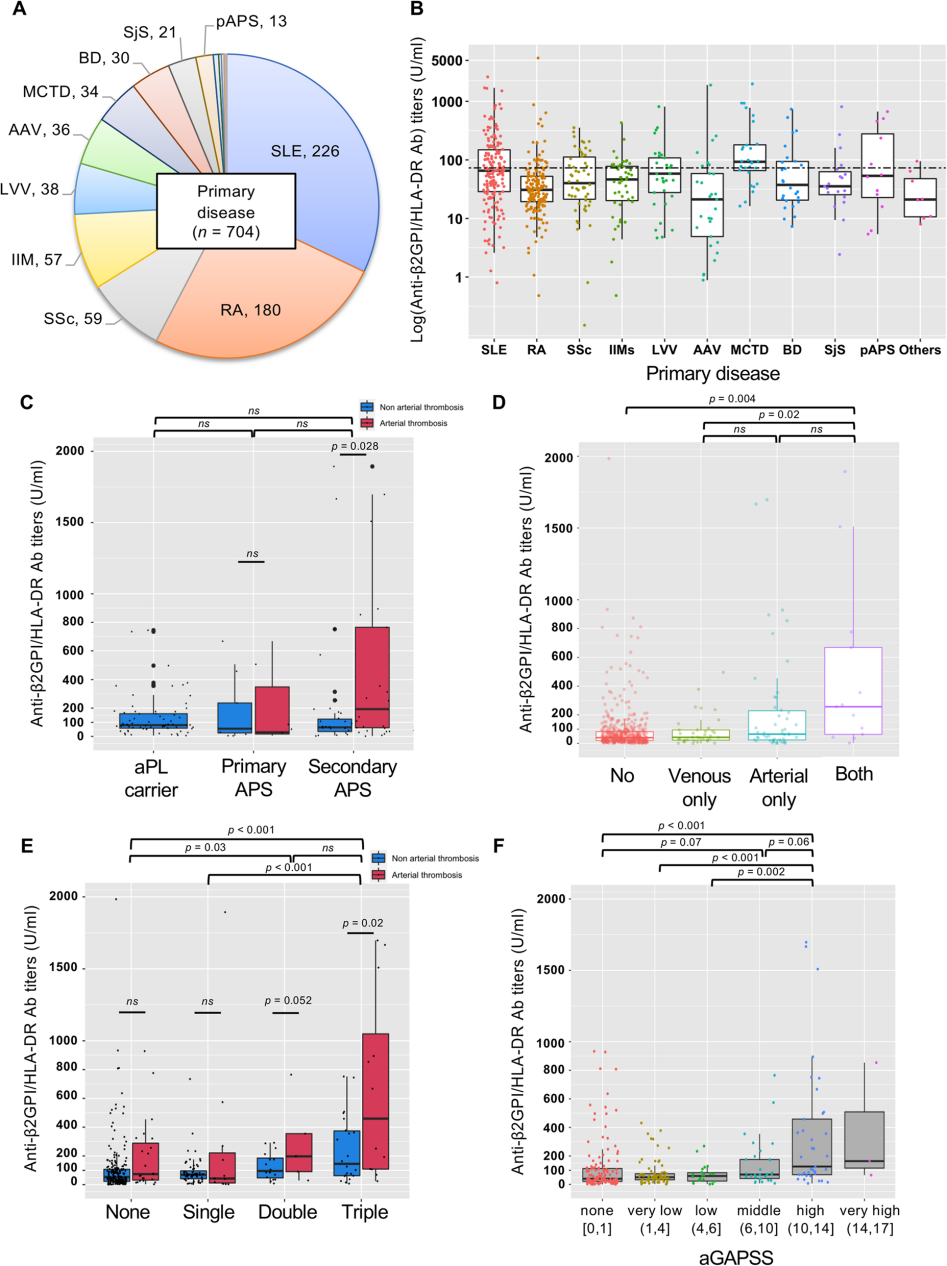
Levels of anti-β2GPI/HLA-DR antibodies in all participants with primary diseases. Abbreviations: *AAV*, ANCA-associated vasculitis; *aGAPSS*, adjusted global APS score; *aCL*, anticardiolipin antibody; *aβ2GPI*, anti-β2GPI antibody; *aPL*, antiphospholipid antibody; *APS*, antiphospholipid antibody syndrome; *BD*, Bechet disease; *IIMs*, idiopathic inflammatory myopathies; *LA*, lupus anticoagulant; *LVV*, large-vessel vasculitis; *pAPS*, primary antiphospholipid antibody syndrome; *RA*, rheumatoid arthritis; *SjS*, Sjögren’s syndrome; *SLE*, systemic lupus erythematosus; *SSc*, systemic sclerosis. **A** Anti-β2GPI/HLA-DR antibodies were quantified in patients with various primary systemic rheumatic diseases. **B** Anti-β2GPI/HLA-DR antibody titers (in U/mL) from each primary disease. The dashed line indicates the cut-off value of 172.359 U/mL, resulting from Fig. 3. A cut-off value of 73.3 U/mL has been reported when comparing APS to healthy participants. **C** Anti-β2GPI/HLA-DR antibody titers among different APS subsets (carrier/primary/secondary). **D** Anti-β2GPI/HLA-DR antibody titers were significantly higher in patients with both arterial and venous thrombosis than in those with no or venous-only thrombosis. **E** Among different aPL subsets (none/single/double/triple-positive), anti-β2GPI/HLA-DR antibody titers increased with the order of increasing aPL positivity. **F** Anti-β2GPI/HLA-DR antibody titers increased with increasing order of aGAPSS clusters: none (< 1 point), very low (1–3 points), low (4–5 points), middle (6–9 points), high (10–13 points), and very high (≥ 14 points)

Figure 1A illustrates the primary diseases among the 704 patients in a clockwise direction of the pie chart. Anti-β2GPI/HLA-DR antibodies among different clinical groups are shown in Fig. 1B–F, and the frequencies of occurrence and details with arterial thrombosis are tabulated in Figure S1B and Table S1. The majority of the systemic rheumatic diseases had a high cut-off value of more than 99th percentile (Fig. 1B). Although patients with primary APS showed no deviations between the two groups, patients with secondary APS showed higher anti-β2GPI/HLA-DR antibody levels in the arterial thrombosis group than those in the non-arterial thrombosis group (Fig. 1C). Anti-β2GPI/HLA-DR antibody titers were significantly higher in patients with both arterial and venous thrombosis than those with no or venous-only thrombosis (Fig. 1D). There were no significant differences between the aPL carrier and definite APS (Figure S2A). The anti-β2GPI/HLA-DR antibody levels were elevated in patients with thrombotic APS, while patients with obstetric APS did not show increased antibody levels compared with those in the aPL carrier (Figure S2B). The more remarkable clusters of aGAPSS or aPL positivity showed higher frequencies of arterial thrombosis and the median of anti-β2GPI/HLA-DR antibodies (Fig. 1E, F). Frequencies of arterial thrombosis with the aGAPSS clusters are depicted in Figure S3.

### The diagnostic value of anti-β2GPI/HLA-DR antibodies

**Fig. 2:**
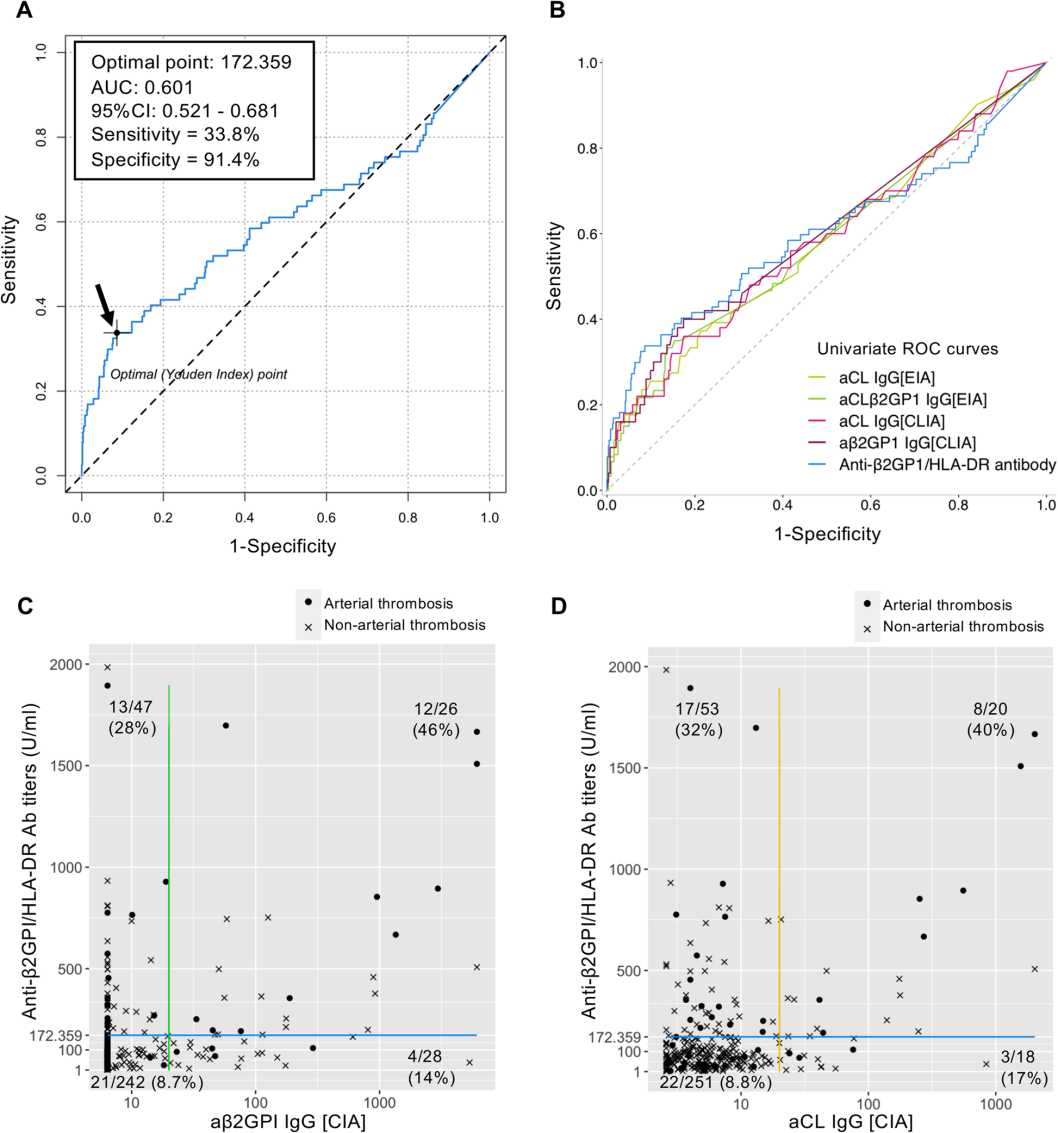
Optimal cut-off and comparison of univariate ROC curve analysis. Abbreviations: *AUC*, area under the curve; *aCL*, anticardiolipin antibody; *aβ2GPI*, anti-β2GPI antibody; CIA, chemiluminescent immunoassay; *ROC*, receiver operating characteristic. **A** Receiver operating characteristics (ROC) analysis for anti-β2GPI/HLA-DR antibodies was performed to determine the most suitable cut-off value that maximized the area under the curve (AUC) between non-arterial thrombosis (*n* = 627) and arterial thrombosis (*n* = 77). The ROC analysis revealed an AUC of 0.601 with a sensitivity and specificity of 33.8% and 91.4%, respectively, at the optimal cut-off level of 172.359 U/mL, using Youden’s index. **B** Five ROC plots of the individual aPLs were compared to each parameter that contains aCL IgG [EIA], aCLβ2GPI IgG [EIA], aCL IgG [CIA], aβ2GPI [CIA], and the anti-β2GPI/HLA-DR antibody. Other differential aPLs are listed in Table 2. The ROC curve for the anti-β2GPI/HLA-DR antibody was the same blue curve seen in **A**. **C**, **D** Comparison between anti-β2GPI/HLA-DR antibody titers and aβ2GPI IgG antibody or aCL IgG antibody titers. The cut-off value of 172.359 U/ml for the anti-β2GPI/HLA-DR antibody was shown as the blue line. The 99th percentile cut-off value of 20 U/ml for aβ2GPI IgG antibody or aCL IgG antibody was shown as the green or yellow line, respectively. The number and percentage of patients with histories of arterial thrombosis among included patients in each quadrant were presented

The ROC curve of the anti-β2GPI/HLA-DR antibody showed a sensitivity, specificity, and AUC for arterial thrombosis of 33.8%, 91.4%, and 0.6009, respectively, with a cut-off value of 172.359 U/mL (Fig. 2A). We assessed the diagnostic performances of aPL antibodies for arterial thrombosis with univariate ROC analysis and Fisher’s exact test (Fig. 2B and Table 2, respectively). The anti-β2GPI/HLA-DR antibody in the 99th percentile had greater sensitivity to aβ2GPI than the other isotype. The anti-β2GPI/HLA-DR antibodies had a plausible ROC curve, and their optimal cut-off (≥ 172.359 U/mL) yielded better accuracy than the other autoantibodies for aPL. From the scatter plot analysis comparing the performance for the detection of arterial thrombosis between anti-β2GPI/HLA-DR antibody and aβ2GPI IgG antibody or aCL IgG antibody (Fig. 2C, D), about 30% of patients who were positive for anti-β2GPI/HLA-DR antibody but aβ2GPI-negative or aCL-negative had histories of arterial thrombosis. Spearman’s rank correlation rho in patients with positive for both antibodies were 0.387 (*p*-value = 0.0508) for anti-β2GPI/HLA-DR antibody and aβ2GPI IgG antibody and 0.559 (*p*-value = 0.0116) for anti-β2GPI/HLA-DR antibody and aCL IgG antibody, respectively. In addition, to assess the potential impact of anti-β2GPI/HLA-DR antibody alone, we extracted patients with all negative aPL profiles (*n* = 203). Twenty-seven patients showed higher titers of anti-β2GPI/HLA-DR antibody than the cut-off value, and 26% of them had histories of arterial thrombotic events. In contrast, among one hundred seventy-six patients with lower titers of anti-β2GPI/HLA-DR antibody than the cut-off value, only 5.7% of them had histories of arterial thrombotic events (*p* = 0.003, Fisher’s exact test).

**Table 2:** Diagnostic performances of aPL antibodies for arterial thrombosis

	Fisher’s exact test												ROC curve analysis (Youden index)								
Parameters	All, *n*	Pos., *n*	Neg., *n*	Sn	Sp	PPV	NPV	Accuracy	LR +	LR −	OR (95% CI)	Cut-off	All, *n*	Pos., *n*	Neg., *n*	Sn	Sp	Accuracy	AUC	Youden’s index	Optimal point
LA test	368	84	284	0.444	0.809	0.286	0.894	0.755	2.326	0.687	3.37 (1.75–6.45)	1.2	−	−	−	−	−	−	−	−	−
aCL IgG [EIA]	304	75	229	0.353	0.775	0.240	0.856	0.704	1.567	0.835	1.87 (0.92–3.72)	10	304	51	253	0.373	0.787	0.717	0.583	0.159	12.0
aCL IgG [CIA]	342	38	304	0.220	0.908	0.289	0.872	0.807	2.379	0.859	2.75 (1.14–6.32)	20	342	50	292	0.360	0.825	0.757	0.587	0.185	10.7
aCL IgM [CIA]	341	18	323	0.080	0.952	0.222	0.858	0.824	1.663	0.966	1.71 (0.39–5.79)	20	341	50	291	0.620	0.533	0.546	0.542	0.153	2.30
aCLβ2GPI IgG [EIA]	378	35	343	0.200	0.928	0.343	0.860	0.812	2.765	0.862	3.19 (1.35–7.22)	3.5	378	60	318	0.350	0.846	0.767	0.584	0.196	1.30
aβ2GPI IgG [CIA]	343	54	289	0.320	0.870	0.296	0.882	0.790	2.467	0.781	3.14 (1.47–6.53)	20	343	50	293	0.826	0.400	0.764	0.602	0.226	14.0
aβ2GPI IgM [CIA]	343	18	325	0.100	0.956	0.278	0.862	0.831	2.254	0.942	2.38 (0.63–7.57)	20	343	50	293	0.911	0.160	0.802	0.510	0.071	7.10
anti-β2GPI/HLA-DR antibody	704	193	511	0.442	0.746	0.176	0.916	0.713	1.741	0.748	2.32 (1.39 − 3.88)	73.3	704	77	627	0.338	0.914	0.851	0.601	0.252	172.359

*Abbreviations: aPL*, antiphospholipid antibody; *APS*, antiphospholipid antibody syndrome; *aCL*, anticardiolipin antibody; *aβ2GPI*, anti-β2GPI antibody; *LA*, lupus anticoagulant; *LR*, likelihood ratio; *OR*, odds ratio; *NPV*, negative predictive value; *PPV*, positive predictive value

Various aPL antibodies measured by EIA or chemiluminescent immunoassay (CIA) were evaluated by calculating the sensitivity, specificity, positive predictive value (PPV), negative predictive value (NPV), accuracy, positive or negative likelihood ratio (LR + or LR −), and odds ratio

### Assessment for conventional cardiovascular risk factors with anti-β2GPI/HLA-DR antibody

Further, we examined the impact of anti-β2GPI/HLA-DR antibodies in the multivariate models.

Table 3 shows the results of the univariable or multivariable logistic regression analysis for arterial thrombosis with complete cases and MI. The VIFs were under five for all independent variables. In multivariate analysis with complete cases, higher age, higher current doses of prednisolone, and anti-β2GPI/HLA-DR antibody levels ≥ 172.359 U/mL were significantly associated with arterial thrombosis. A high pack-year smoking index and a proportion of arterial hypertension were adequately related to arterial thrombosis in cases with MI.

**Table 3:** Univariable and multivariable logistic regression analysis with multiple imputation

	Univariate		Multivariate					
			Complete cases (*n* = 409)				Multiple imputation (*n* = 704)	
			Conventional cardiovascular risk factors alone (model 1)		The addition of anti-β2GPI/HLA-DR antibody status (model 2)		The addition of anti-β2GPI/HLA-DR antibody status (model 2)	
Variables	OR (95%CI)	*p*-value	OR (95%CI)	*p*-value	OR (95%CI)	*p*-value	OR (95%CI)	*p*-value
Age	1.02 (1.00–1.03)	0.050	1.02 (1.00–1.04)	0.043	1.03 (1.00–1.05)	0.027	1.01 (0.99–1.03)	0.059
Disease duration	1.01 (0.99–1.03)	0.341	1.00 (0.98–1.03)	0.790	1.00 (0.98–1.03)	0.850	1.00 (0.98–1.02)	0.610
Body mass index	1.04 (0.97–1.10)	0.270	1.02 (0.95–1.09)	0.651	1.01 (0.94–1.09)	0.730	1.01 (0.94–1.08)	0.750
Pack-year smoking	1.04 (1.02–1.07)	0.002	1.03 (1.00–1.07)	0.063	1.03 (1.00–1.07)	0.073	1.04 (1.01–1.07)	0.007
Current doses of prednisolone	1.07 (1.01–1.13)	0.014	1.11 (1.02–1.20)	0.012	1.11 (1.03–1.20)	0.009	1.07 (1.01–1.13)	0.018
Dyslipidemia	1.13 (0.68–1.89)	0.632	0.73 (0.35–1.49)	0.387	0.78 (0.37–1.61)	0.500	0.77 (0.42–1.40)	0.401
Arterial hypertension	2.54 (1.56–4.16)	< 0.001	1.82 (0.93–3.58)	0.081	1.81 (0.91–3.61)	0.092	2.37 (1.33–4.23)	0.003
Diabetes	1.62 (0.83–3.17)	0.155	1.04 (0.42–2.56)	0.932	1.26 (0.51–3.12)	0.620	1.15 (0.52–2.52)	0.723
Anti-β2GPI/HLA-DR antibody status (cut-off = 172.359 U/ml)	5.10 (2.94–8.87)	< 0.001	—	—	4.39 (2.14–9.03)	< 0.001	5.13 (2.85–9.24)	< 0.001

*Abbreviations: CI*, confidence interval; *OR*, odds ratio

The variance inflation factors were < 5 for all of the independent variables. Compared with the non-arterial thrombosis group, the arterial thrombosis group had a significantly positive anti-β2GPI/HLA-DR antibody cut-off (odds ratio: 5.13; 95% CI: 2.85−9.24) in multivariate logistic regression analysis with multiple imputation. Increased age, a high prednisolone dose, high pack-year smoking, and a history of arterial hypertension were significantly associated with arterial thrombosis

The odds ratio for anti-β2GPI/HLA-DR antibody levels ≥ 172.359 U/mL was 5.13 (95% CI: 2.85–9.24) for arterial thrombosis in multivariate analysis with MI. Disease duration, BMI, dyslipidemia, and diabetes were not linked to arterial thrombosis in this study.

### Improvement in multivariate models with anti-β2GPI/HLA-DR antibody

**Fig. 3:**
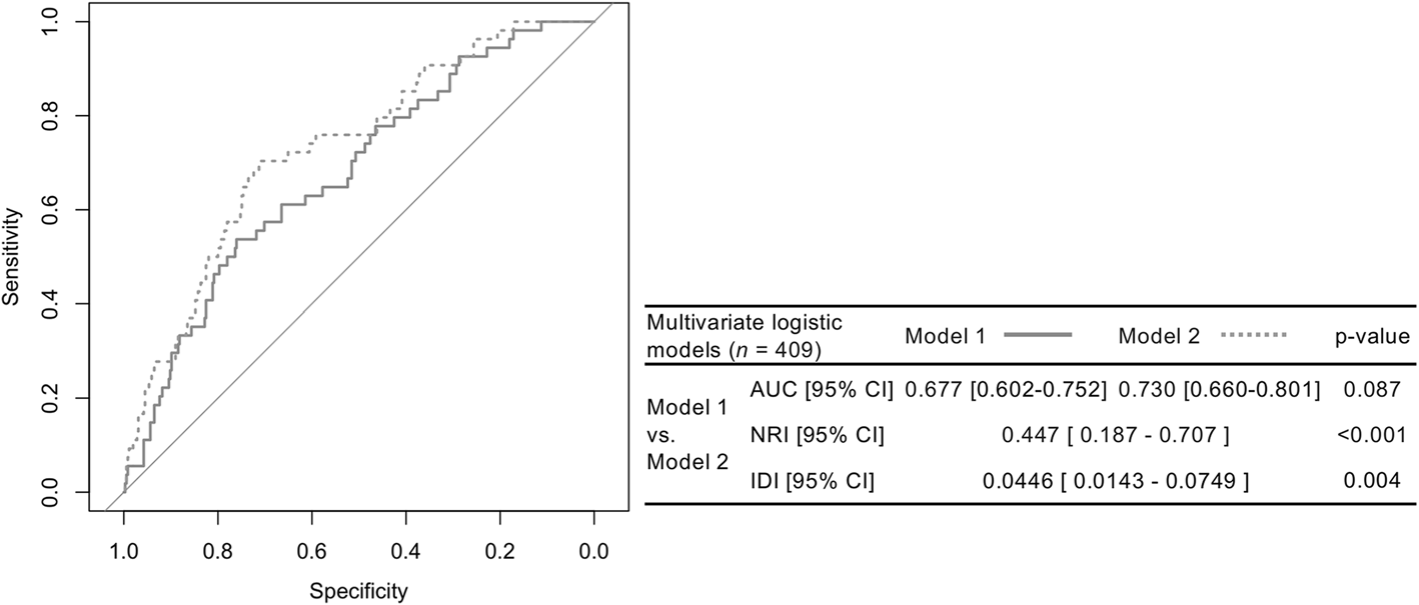
Comparison of ROC curves between multivariate logistic regression models and reclassification analysis. Abbreviations: *ROC*, receiver operating characteristic; *AUC*, area under the curve; *NRI*, net reclassification improvement; *IDI*, integrated discrimination improvement; *CI*, confidence interval; *OR*, odds ratio. Comparison between two models in multivariate logistic regression analysis with complete cases (*n* = 409). Variables for model 1; conventional cardiovascular risk factors contain age, disease duration, body mass index, smoking habit, the current dose of prednisolone, dyslipidemia, arterial hypertension, and diabetes. Variables for model 2; adding anti-β2GPI/HLA-DR antibody cut-off to model 1. There was no significant difference with C-index improvement for the detection of arterial thrombosis, but continuous NRI and IDI were statistically significant

We compared two multivariate and multiclass ROC analyses (Table 3). These AUCs (Fig. 3) were used to evaluate and compare the discrimination efficacy of the multivariate models.

To detect arterial thrombotic events, anti-β2GPI/HLA-DR antibody cut-off to the conventional cardiovascular risks improved the AUCs (model 1 vs. model 2; 0.677 to 0.730). Unfortunately, there were no significant differences (model 1 vs. model 2; *p* = 0.087) with C-index improvement. Further analyses with continuous NRI (model 1 vs. model 2; 0.447 [95% CI: 0.187–0.707], *p*-value < 0.001) and IDI (model 1 vs. model 2; 0.0446 [95% CI: 0.0143–0.0749], *p*-value = 0.004) were statistically significant. The calibration plots showed the fitness of these two models (Figure S4). The addition of anti-β2GPI/HLA-DR antibody cut-off (model 2) showed discrimination and a better improvement of calibration than the model of conventional cardiovascular risk factors alone (model 1).

## Discussion

To the best of our knowledge, this is the first study to elucidate the association between anti-β2GPI/HLA-DR antibodies and arterial thrombotic episodes in female patients with various systemic rheumatic diseases.

In regard to venous thromboses, many risk factors, such as age, immobility, dehydration, obesity, pregnancy, cancer, surgery, and anti-tumor drugs or estrogen therapy [32], vary considerably from those of arterial thrombosis and are sometimes transient. Hence, it was difficult to establish the association between anti-β2GPI/HLA-DR antibodies and venous thrombosis from this cross-sectional study. Above all, β2GPI-dependent aCL and anti-β2GPI antibodies are reported to be key predictors of arterial thrombosis [33, 34]. Thus, this study focused on arterial thrombosis.

Moreover, Japanese APS patients are likely to have the predominant type of arterial thrombosis [35]. Most arterial thromboses in APS are cerebral vasculature, usually in the form of a stroke or TIA [36]. Similarly, our study population also showed an equivalent distribution of arterial thrombosis.

Interestingly, approximately one-third of the study participants showed anti-β2GPI/HLA-DR antibody titers above the 99th percentile calculated from healthy controls.

Although SLE or MCTD, wherein the HLA-DRs are related to disease susceptibility, tend to have high titers [37–39], HLA-DR variations alone could not justify this finding. The disease-susceptible MHC class II alleles of APS (HLA-DRB1*04, HLA-DRB1*07) are efficiently expressed on the cell surface in combination with non-peptide β2GPI and recognized by aPL. Simultaneously, almost all HLA-DRs, except HLA-DRB1*04 and HLA-DRB1*07, also express β2GPI and bind to aPL [7, 40]. Although we could not show evidence of the study patient’s MHC class II, its heterogeneity in background diseases influenced the variation in anti-β2GPI antibodies among various systemic rheumatic diseases.

Particularly, pro-inflammatory cytokines, such as IFNγ and TNFα, are important for MHC class II expression on non-antigen-presenting cells [41, 42]. In addition, IFNγ and TNFα promote the β2GPI/HLA-DR expression on endothelial cells [7]. The type I IFN signature affects the pathogenesis of several rheumatic autoimmune diseases, including SLE, RA, SSc, IIMs, and APS [43, 44]. Type I IFN upregulation also affects the increased production of circulating plasmablasts linked to aPL [45]. Thus, the upregulation of various cytokines, including type I IFN, in various primary diseases could explain the high titers of anti-β2GPI/HLA-DR antibodies.

Additionally, the anti-β2GPI/HLA-DR antibody titers were increased in the subset with arterial thrombotic episodes and were more frequent and significantly higher in patients with triple-positive or aGAPSS > 10, which were previously reported to be at an increased risk for arterial thrombosis. IFNγ or TNFα itself is crucial in accelerating inflammatory atherogenesis. Moreover, anti-β2GPI antibodies themselves may increase vascular inflammation and enhance atherosclerosis [46]. Anti-β2GPI antibodies are also significantly associated with the thickness of the intima-media [47]. Anti-β2GPI/HLA-DR antibodies induced on these inflammatory sites and mediated pro-inflammatory atherothrombosis may demonstrate pathogenic mechanisms of cardiovascular events in systemic rheumatic diseases. Hence, the anti-β2GPI/HLA-DR antibody can indicate the quantitative risk for arterial thrombosis, which may aid in calculating the reasonable cut-off value using univariate ROC analysis.

Unfortunately, all primary APS patients included in this study were only thirteen, and only four of those patients were classified into the arterial thromboses group. Therefore, we could not discuss whether the difference in the titers of anti-β2GPI/HLA-DR antibodies between the arterial thrombosis group and the non-arterial thrombosis group were specific in secondary APS but not in primary APS. In the bivariate analysis, the higher current and maximum doses of glucocorticoids are expected to be higher cumulative doses, suggesting higher disease activity in the arterial thrombus group. Although the titers of anti-DNA (anti-dsDNA) antibodies and the positivity of the other types of autoantibodies were not different in both groups, higher anti-β2GPI/HLA-DR antibody levels may reflect an over-expressed type I IFN signature and B-cell responses based on disease activity in patients with histories of arterial thrombosis.

In general, arterial thrombosis is frequent in triple-positive cases with high β2GPI IgG levels. No thrombosis with aPL carriers is considered a pre-thrombotic phase and needs reevaluation as a trigger for the development of thrombotic APS [48]. These triggers include reported infection, smoking, long-term immobility, pregnancy, oral contraceptive, malignancy, nephrosis, arterial hypertension, and hyperlipidemia [49]. In addition, the higher complication rates of classical cardiovascular risk factors in the arterial thrombosis group may also derive from the higher disease activity and cumulative glucocorticoid doses. Therefore, it is necessary to investigate the independent performance of anti-β2GPI/HLA-DR antibodies in a combination of generic cardiovascular risk factors. Accordingly, we confirmed the clinical benefit of setting an anti-β2GPI/HLA-DR antibody cut-off to the conventional cardiovascular risks in a multivariate model. The positivity of the anti-β2GPI/HLA-DR antibody was the independent factor and showed the highest odds ratio among several factors, including the current dosage of glucocorticoids.

It is well known that the significance may be underestimated in the difference with C-index improvement by the Delong methods [50]. Therefore, we evaluated the added predictive ability of a new marker by calculating continuous NRI and IDI analyses [51]. As a result, we considered that anti-β2GPI/HLA-DR antibodies yielded significant discrimination found in Fig. 3. However, the predictive performance for arterial thrombosis should be checked by internal validation [52]. The odds ratio in the anti-β2GPI/HLA-DR antibody cutoff of 4.39 (95%CI: 2.14–9.03) showed discrimination and a good improvement of calibration.

By using sera from patients with the diagnosis of APS, our previous study showed that anti-β2GPI/HLA-DR antibody was more sensitive than aβ2GPI IgG antibody or aCL IgG antibody. At the same time, the titers of anti-β2GPI/HLA-DR antibody and aβ2GPI IgG antibody or aCL IgG antibody were well-correlated in patients positive for both antibodies [7]. In the present study of female patients with various autoimmune diseases, the serum samples in patients with positive for both antibodies had also a correlation between the anti-β2GPI/HLA-DR antibody titers and anti-β2GPI IgG antibody or aCL IgG antibody titers. Furthermore, the proportions of patients with histories of arterial thrombosis in the anti-β2GPI/HLA-DR antibody alone-positive population were higher than those in aβ2GPI IgG antibody or aCL IgG antibody alone-positive population. Anti-β2GPI/HLA-DR antibody above the cut-off value could detect patients with histories of arterial thrombosis, even in aPL-negative cases. These results are similar to those of our previous study, which reported that anti-β2GPI/HLA-DR antibodies could detect shared epitopes with β2GPI complexes formed with either cardiolipin or negatively charged plates while recognizing the other unique epitopes [7].

Simultaneously, our results implied that high blood pressure, high amounts of smoking, increased age, and high daily glucocorticoid doses could help screen arterial thrombotic risk. Similarly, Erkan et al. reported high blood pressure and smoking as risk factors for arterial thrombosis [49]. Cumulative and higher daily glucocorticoid doses are considered clinical predictors of thrombosis or atherosclerosis in patients with SLE [53, 54]. EULAR recommendations for cardiovascular risk have indicated glucocorticoid dose minimization in SLE and vasculitis [55].

In some clinical studies, the global antiphospholipid syndrome score (GAPSS) is often applied as the scoring scale to identify the increased risk of thrombosis or APS. aGAPSS, which excludes aPS/PT antibody, is more straightforward than GAPSS. Yet, the glucocorticoid dose and the amount of smoking are not included in aGAPSS. We should consider these as risk factors for arterial thrombosis due to a lack of validated rheumatic disease-specific scales for cardiovascular risk, even if there is a small odds ratio [56].

In this study, we identified the clinical decision limit at 172.359 U/mL for arterial thrombosis. Most clinicians must verify if thromboses are related to APS because some patients require persistent prophylaxis in cases with severe, multiple, or recurrent thromboses. When the difference between the diagnostic and treatment threshold can be distinguished in these titers, this cut-off (≥ 172.359 U/mL) may guide clinical practice for improving cardiovascular risk management.

The limitations of this study are as follows. First, although patients in this study were tested for aPL, all patients were not confirmed for the LA test using the diluted Russell’s viper venom time, since multiple simultaneous tests are not accepted by medical insurance in Japan. Second, only female participants were included in this study. Male patients are known to be at high risk of thrombotic recurrence [57]. Third, the number of patients with arterial thrombosis was insufficient to establish firm conclusions from the multivariate logistic regression analysis with complete cases. Less than half of patients have been tested for classical APS antibodies, and complements also have less than half of the patient data. Imputation analysis on these values might be inaccurate. Fourth, this study was based on medical records and questionnaires; there may be non-respondent and recall bias. So, the titers of anti-β2GPI/HLA-DR antibodies in the group of obstetric APS may not have shown increased levels compared with those in the aPL carrier. Fifth, we could not confirm the onset time of thrombosis. The values of anti-β2GPI/HLA-DR antibodies may have already decreased because the samples were not collected in the active thrombotic phase.

## Conclusions

In conclusion, we identified that the anti-β2GPI/HLA-DR antibodies were related to arterial thrombosis in female patients with systemic rheumatic diseases. Considering that risk stratification is essential, the anti-β2GPI/HLA-DR antibody may help suggest arterial thrombosis, even though they coexist with conventional cardiovascular risk factors, and determine the administration of anticoagulants. Nonetheless, the performance of anti-β2GPI/HLA-DR antibodies should be confirmed in a further prospective investigation for external validation.

### Supplementary Information

**Additional file 1: Figure S1**. (A) Flow diagram of the study design and (B) pie chart of arterial thrombosis. A All female patients visited the department of Rheumatology and Clinical Immunology at Kobe University Hospital from April 2020 to December 2021. Of these, 721 patients consented to the study, and 704 provided blood samples and questionnaires. Among the 704 patients, 121 reported one or more events of thrombosis. A history of arterial thrombosis was reported in 77 patients, and 14 had both arterial and venous thromboses. B The pie chart shows arterial thrombotic episodes as a number per manifestation. The most common manifestation was cerebral infarction. Digital vascular complications included finger thrombosis or gangrenes, excluding digital ulceration related to scleroderma diagnosed by clinicians. Abdominal arterial thrombosis includes thrombosis involving the abdominal aorta and the branch. **Figure S2.** Anti-β2GPI/HLA-DR antibody within each group. Abbreviations: *aPL*, antiphospholipid antibody; *APS*, Antiphospholipid antibody syndrome. A Anti-β2GPI/HLA-DR antibody titers in patients with no aPL (LA, aCL, and aβ2GPI were all negative), aPL carrier, and APS who fulfilled the criteria. B Anti-β2GPI/HLA-DR antibody titers with variants of APS, including thrombotic APS (tAPS), obstetric APS (oAPS), both thrombotic and obstetric APS (t + oAPS), and aPL carrier (no thrombotic or obstetric comorbidities related to APS). **Figure S3.** Frequency of arterial thrombosis within each cluster of aGAPSS. Abbreviations: *aGAPSS*, adjusted global APS score. The cluster of aGAPSS: none (< 1 point), very low (1–3 points), low (4–5 points), middle (6–9 points), high (10–13 points), very high (≥ 14 points). **Figure S4.** Reliability diagram. A graph of the observed frequency of arterial thrombotic events plotted against the score values obtained by the predictive model in the multivariate logistic regression analyses. This graph is often used for calibration visualization. The horizontal axis shows the mean predicted score value, and the vertical axis shows the proportion of positive labels for arterial thrombotic events. An extensive dashed line for the situation in which predicted probabilities perfectly match the observed probabilities is drawn as an ideal. Bootstrapping using 300 repetitions was used to get the bias-corrected curve of the predicted versus the actual probability. The apparent and bias-corrected curves got closer to the ideal line in model 2 than in model 1.

**Additional file 2: Table S1.** Details of thrombosis episodes and aPL categories.

## Data Availability

JS had complete access to all of the raw data in the study and took responsibility for the integrity of the data and the accuracy of the data analysis. Data are available upon reasonable request.

## Abbreviations

aβ2GPI: Anti-β2-glycoprotein I
HLA-DR: Human leukocyte antigen DR
APS: Antiphospholipid syndrome
aGAPSS: Adjusted global antiphospholipid syndrome score
ROC: Receiver operating characteristic
AUC: Area under the curve
aPL: Anti-phospholipid antibody
aCL: Anti-cardiolipin
ELISA: Enzyme-linked immunosorbent assay
MHC: Major histocompatibility complex
IFNγ: Interferon-gamma
TNFα: Tumor necrosis factor-alpha
AMED: Japan Agency for Medical Research and Development
SLE: Systemic lupus erythematosus (SLE)
ACR: American College of Rheumatology
MCTD: Mixed connective tissue disease
SSc: Systemic sclerosis
EULAR: European Alliance of Associations for Rheumatology
SjS: Sjögren’s syndrome
IIMs: Inflammatory myositis
UCTD: Unclassified connective tissue disease (UCTD)
BMI: Body mass index (BMI)
CT: Computed tomography
MRI: Magnetic resonance imaging (MRI)
TIA: Transient ischemic attack
CIA: Chemiluminescent immunoassay
aCLβ2GPI: Anti-cardiolipin β2 glycoprotein I
LA: Lupus anticoagulant
aPS/PT: Anti-phosphatidylserine-prothrombin
VIF: Variance inflation factor
NRI: Net reclassification improvement
IRI: Integrated discrimination improvement
CI: Confidence interval
MI: Multiple imputation
MICE: Multiple imputation by chained equation
BD: Bechet disease
LVV: Large-vessel vasculitis
pAPS: Primary Antiphospholipid antibody syndrome
RA: Rheumatoid arthritis
CRP: C-reactive protein
EIA: Enzyme immunoassay
IQR: Interquartile range
PLT: Platelet
SD: Standard deviation
LR: Likelihood ratio
OR: Odds ratio
NPV: Negative predictive value
PPV: Positive predictive value
AZA: Azathioprine
csDMARDs: Conventional synthetic disease-modifying anti-rheumatic drugs
CNS: Central nervous system
HCQ: Hydroxychloroquine
IGU: Iguratimod
IL-6R: Interleukin-6 receptor
IS: Immunosuppressant
IVCY: Intravenous cyclophosphamide
IVIG: Intravenous immunoglobulin
LEF: Leflunomide
MMF: Mycophenolate mofetil
mPSL: Methylprednisolone
MTX: Methotrexate
MZR: Mizoribine
POCY: Per oral cyclophosphamide
SASP: Salazosulfapyridine
TAC: Tacrolimus
JAK: Janus kinase
