# Supplementary material for: Association of anti-β2-glycoprotein I/HLA-DR complex antibody with arterial thrombosis in female patients with systemic rheumatic diseases
Source: Arthritis Res Ther. 2023 Oct 6;25:195. doi: 10.1186/s13075-023-03175-8 (PMC10557208; doi:10.1186/s13075-023-03175-8)
Supplement: Supplementary file 1 — Additional file 1: Figure S1. (A) Flow diagram of the study design and (B) pie chart of arterial thrombosis. A All female patients visited the department of Rheumatology and Clinical Immunology at Kobe University Hospital from April 2020 to December 2021. Of these, 721 patients consented to the study, and 704 provided blood samples and questionnaires. Among the 704 patients, 121 reported one or more events of thrombosis. A history of arterial thrombosis was reported in 77 patients, and 14 had both arterial and venous thromboses. B The pie chart shows arterial thrombotic episodes as a number per manifestation. The most common manifestation was cerebral infarction. Digital vascular complications included finger thrombosis or gangrenes, excluding digital ulceration related to scleroderma diagnosed by clinicians. Abdominal arterial thrombosis includes thrombosis involving the abdominal aorta and the branch. Figure S2. Anti-β2GPI/HLA-DR antibody within each group. Abbreviations: aPL, antiphospholipid antibody; APS, Antiphospholipid antibody syndrome. A Anti-β2GPI/HLA-DR antibody titers in patients with no aPL (LA, aCL, and aβ2GPI were all negative), aPL carrier, and APS who fulfilled the criteria. B Anti-β2GPI/HLA-DR antibody titers with variants of APS, including thrombotic APS (tAPS), obstetric APS (oAPS), both thrombotic and obstetric APS (t + oAPS), and aPL carrier (no thrombotic or obstetric comorbidities related to APS). Figure S3. Frequency of arterial thrombosis within each cluster of aGAPSS. Abbreviations: aGAPSS, adjusted global APS score. The cluster of aGAPSS: none (< 1 point), very low (1–3 points), low (4–5 points), middle (6–9 points), high (10–13 points), very high (≥ 14 points). Figure S4. Reliability diagram. A graph of the observed frequency of arterial thrombotic events plotted against the score values obtained by the predictive model in the multivariate logistic regression analyses. This graph is often used for calibration visualization. The horizont [file 13075_2023_3175_MOESM1_ESM.docx]

**Supporting information**

**Supplementary figures**

**Figure S1.** (A) Flow diagram of the study design and (B) pie chart of arterial thrombosis.

1. All female patients visited the department of Rheumatology and Clinical Immunology at Kobe University Hospital from April 2020 to December 2021. Of these, 721 patients consented to the study, and 704 provided blood samples and questionnaires. Among the 704 patients, 121 reported one or more events of thrombosis. A history of arterial thrombosis was reported in 77 patients, and 14 had both arterial and venous thromboses.
2. The pie chart shows arterial thrombotic episodes as a number per manifestation. The most common manifestation was cerebral infarction. Digital vascular complications included finger thrombosis or gangrenes, excluding digital ulceration related to scleroderma diagnosed by clinicians. Abdominal arterial thrombosis includes thrombosis involving the abdominal aorta and the branch.

**Figure S2.** Anti-β2GPⅠ/HLA-DR antibody within each group.


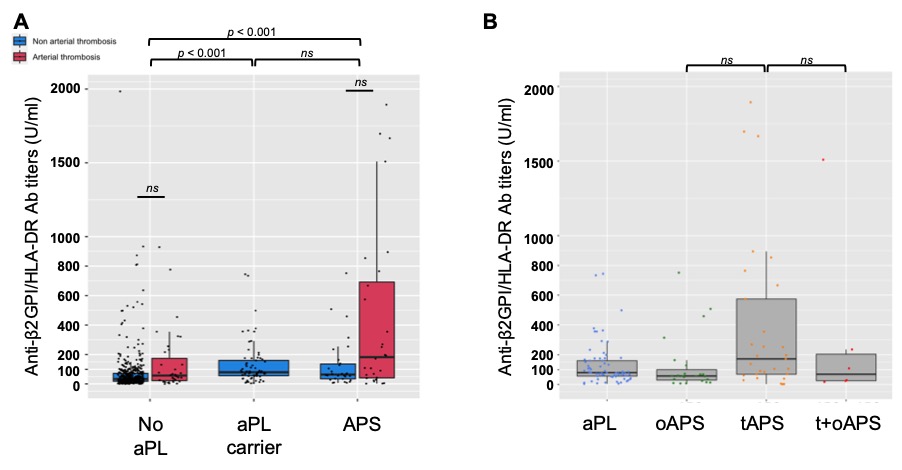


Abbreviations: *aPL*, antiphospholipid antibody; *APS*, Antiphospholipid antibody syndrome.

1. Anti-β2GPⅠ/HLA-DR antibody titers in patients with no aPL (LA, aCL, and aβ2GPⅠ were all negative), aPL carrier, and APS who fulfilled the criteria.
2. Anti-β2GPⅠ/HLA-DR antibody titers with variants of APS, including thrombotic APS (tAPS), obstetric APS (oAPS), both thrombotic and obstetric APS (t+oAPS), and aPL carrier (no thrombotic or obstetric comorbidities related to APS).

**Figure S3.** Frequency of arterial thrombosis within each cluster of aGAPSS.


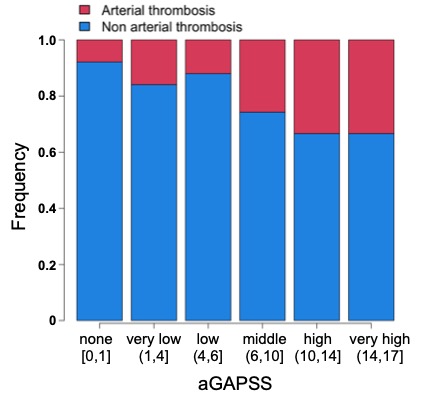


Abbreviations: *aGAPSS*, adjusted global APS score.

The cluster of aGAPSS: none (< 1 point), very low (1–3 points), low (4–5 points), middle (6–9 points), high (10–13 points), very high (≥ 14 points).

**Figure S4.** Reliability diagram

A graph of the observed frequency of arterial thrombotic events plotted against the score values obtained by the predictive model in the multivariate logistic regression analyses. This graph is often used for calibration visualization. The horizontal axis shows the mean predicted score value, and the vertical axis shows the proportion of positive labels for arterial thrombotic events. An extensive dashed line for the situation in which predicted probabilities perfectly match the observed probabilities is drawn as an ideal. Bootstrapping using 300 repetitions was used to get the bias-corrected curve of the predicted versus the actual probability. The apparent and bias-corrected curves got closer to the ideal line in model 2 than in model 1.
