# Supplementary material for: Association of anti-β2-glycoprotein I/HLA-DR complex antibody with arterial thrombosis in female patients with systemic rheumatic diseases
Source: Arthritis Res Ther. 2023 Oct 6;25:195. doi: 10.1186/s13075-023-03175-8 (PMC10557208; doi:10.1186/s13075-023-03175-8)
Supplement: Supplementary file 2 — Additional file 2: Table S1. Details of thrombosis episodes and aPL categories. [file 13075_2023_3175_MOESM2_ESM.docx]

**Supplementary tables**

**Table S1.** Details of thrombosis episodes and aPL categories.

| Variables |  | Thrombosis | | p-value*^2^* |
| --- | --- | --- | --- | --- |
|  | Overall | Non-arterial | Arterial |  |
|  | all = 704*^1^* | n = 627*^1^* | n = 77*^1^* |  |
| aPL categories |  |  |  |  |
| Triple-positive, n (%) | 36 (5.1%) | 23 (3.7%) | 13 (17%) | < 0.001 |
| Double positive, n (%) | 26 (3.6%) | 20 (3.2%) | 6 (7.8%) | 0.054 |
| aCL+aβ2GPI | 17 (2.4%) | 12 (1.9%) | 5 (6.5%) | 0.030 |
| aCL+LA test | 4 (0.6%) | 4 (0.6%) | 0 (0%) | 1.000 |
| aβ2GPI+LA test | 5 (0.7%) | 4 (0.6%) | 1 (1.3%) | 0.439 |
| Single positive, n (%) | 82 (1.2%) | 67 (1.1%) | 15 (1.9%) | 0.036 |
| aCL | 35 (5.0%) | 30 (4.8%) | 5 (6.5%) | 0.574 |
| aβ2GPI | 8 (1.1%) | 8 (1.3%) | 0 (0%) | 1.000 |
| LA test | 39 (5.5%) | 29 (4.6%) | 10 (13%) | 0.006 |
| Negative, n (%) | 203 (29%) | 186 (30%) | 17 (22%) | 0.184 |
| Others, n (%) | 94 (13%) | 82 (13%) | 12 (16%) | 0.593 |
| Negative, except for aCL | 14 (2.0%) | 12 (1.9%) | 2 (2.6%) | 0.659 |
| Negative, except for aβ2GPI | 1 (0.1%) | 1 (0.2%) | 0 (0%) | 1.000 |
| Negative, except for LA test | 66 (9.4%) | 58 (9.3%) | 8 (10%) | 0.682 |
| Negative, except for LA test+aCL | 2 (0.3%) | 1 (0.2%) | 1 (1.3%) | 0.207 |
| Negative, except for aCL+aβ2GPI | 11 (1.6%) | 10 (1.6%) | 1 (1.3%) | 1.000 |
| Unknown, n (%) | 263 (37%) | 249 (40%) | 14 (18%) | < 0.001 |
| Clinical phenotypes in primary and secondary APS patients |  |  |  |  |
| Isolated obstetric APS, n (%) | 23 (3.2%) | 23 (3.3%) | 0 (0%) | 1.000 |
| Isolated thrombotic APS, n (%) | 34 (4.8%) | 8 (1.3%) | 26 (34%) | < 0.001 |
| Both obstetric & thrombotic APS, n (%) | 9 (1.3%) | 1 (0.2%) | 8 (10%) | < 0.001 |
| Vascular thrombotic episodes |  |  |  |  |
| Arterial thrombosis, n (%) |  |  |  |  |
| Coronary heart disease | 18 (2.5%) | 0 (0%) | 18 (23%) | 1.000 |
| Angina | 8 (1.1%) | 0 (0%) | 8 (10%) | 1.000 |
| Myocardial infarction | 10 (1.4%) | 0 (0%) | 10 (12%) | 1.000 |
| Cerebral infarction | 47 (6.7%) | 0 (0%) | 47 (61%) | 1.000 |
| Single-focal infarction | 43 (6.1%) | 0 (0%) | 43 (55%) | 1.000 |
| Multifocal infarction | 2 (0.3%) | 0 (0%) | 2 (2.5%) | 1.000 |
| Recurrent infarction | 2 (0.3%) | 0 (0%) | 2 (2.5%) | 1.000 |
| Digital vascular complications ^a^ | 5 (0.7%) | 0 (0%) | 5 (6.5%) | 1.000 |
| Abdominal arterial thrombosis ^b^ | 1 (0.1%) | 0 (0%) | 1 (1.3%) | 1.000 |
| Lower limb arterial thrombosis | 4 (0.6%) | 0 (0%) | 4 (5.2%) | 1.000 |
| Retinal artery thrombosis | 2 (0.3%) | 0 (0%) | 2 (2.6%) | 1.000 |
| Venous thrombosis, n (%) |  |  |  |  |
| Venous thrombus embolism ^c^ | 50 (7.1%) | 36 (5.7%) | 14 (18%) | < 0.001 |
| Cervical venous thrombosis | 1 (0.1%) | 1 (0.2%) | 0 (0%) | 1.000 |
| Atrial thrombosis | 1 (0.1%) | 1 (0.2%) | 0 (0%) | 1.000 |
| Retinal venous thrombosis | 7 (1.0%) | 7 (1.1%) | 0 (0%) | 1.000 |
| Obstetric episodes |  |  |  |  |
| Obstetric events, n (%) |  |  |  |  |
| 3 or more times recurrent miscarriages at < 10^th^ weeks | 14 (1.9%) | 13 (1.8%) | 1 (0.1%) | 1.000 |
| Fetal death at > 10^th^ weeks | 29 (4.1%) | 26 (3.6%) | 3 (0.4%) | 1.000 |
| Premature birth < 34^th^ weeks ^d^ | 23 (3.2%) | 19 (2.6%) | 4 (0.5%) | 0.305 |
| Non-criteria manifestations |  |  |  |  |
| Extra-criteria APS symptoms, n (%) |  |  |  |  |
| Immune-related thrombocytopenia | 49 (6.9%) | 40 (6.4%) | 9 (11.7%) | 0.095 |
| Seizure | 8 (1.1%) | 3 (0.4%) | 5 (0.7%) | < 0.001 |
| Non-stroke CNS manifestation | 14 (1.9%) | 11 (1.5%) | 3 (0.4%) | 0.190 |
| Immunosuppressant (IS), csDMARDs, or biologics |  |  |  |  |
| IS or csDMARDs |  |  |  |  |
| Currently on AZA, n | 41 (5.82) | 35 (5.58) | 6 (7.79) | 0.437 |
| Currently on MMF, n | 37 (5.25) | 33 (5.26) | 4 (5.19) | 1.000 |
| Currently on MZR, n | 3 (0.42) | 3 (0.47) | 0 (0) | 1.000 |
| Currently on TAC, n | 81 (11.5) | 72 (11.4) | 9 (11.6) | 1.000 |
| Currently on CsA, n | 13 (1.84) | 12 (1.91) | 1 (1.29) | 1.000 |
| Currently on MTX, n | 124 (17.6) | 113 (18.0) | 11 (14.2) | 0.526 |
| Currently on LEF, n | 0 (0) | 0 (0) | 0 (0) | 1.000 |
| Currently on SASP, n | 46 (6.53) | 43 (6.85) | 3 (3.89) | 0.504 |
| Currently on HCQ, n | 55 (7.81) | 49 (7.81) | 6 (7.79) | 1.000 |
| Currently on IGU, n | 30 (4.26) | 28 (4.46) | 2 (2.59) | 0.763 |
| Combination of IS/csDMARDs, n (%) |  |  |  |  |
| None | 375 (53.2) | 333 (53.1) | 42 (54.5) | 0.567 |
| Single | 222 (31.5) | 195 (31.1) | 27 (35.0) |  |
| Dual | 89 (12.6) | 81 (12.9) | 8 (10.3) |  |
| Triple | 17 (2.41) | 17 (2.71) | 0 (0) |  |
| Quadruple | 1 (0.14) | 1 (0.15) | 0 (0) |  |
| Biologics therapy |  |  |  |  |
| Currently on anti TNFα inhibitor, n (%) | 37 (5.25) | 36 (5.74) | 1 (1.29) | 0.169 |
| Currently on anti-IL-6R agents, n (%) | 24 (3.40) | 21 (3.34) | 3 (3.89) | 0.739 |
| Currently on Abatacept, n (%) | 22 (3.12) | 19 (3.03) | 3 (3.89) | 0.724 |
| Currently on JAK inhibitor, n (%) | 18 (2.55) | 18 (2.87) | 0 (0) | 1.000 |
| Currently on Belimumab, n (%) | 9 (1.27) | 8 (1.27) | 1 (1.29) | 1.000 |
| Currently on Rituximab, n (%) | 1 (0.14) | 1 (0.15) | 0 (0) | 1.000 |
| Past history of treatment |  |  |  |  |
| History of mPSL pulse therapy, n (%) | 106 (15.0) | 90 (14.3) | 16 (20.7) | 0.373 |
| History of IVCY pulse or POCY therapy, n (%) | 53 (7.52) | 44 (7.01) | 9 (11.6) | 0.166 |
| History of Plasma exchange, n (%) | 10 (1.42) | 7 (1.11) | 3 (3.89) | 0.085 |
| History of IVIG therapy, n (%) | 12 (1.70) | 12 (1.91) | 0 (0) | 1.000 |
| History of Rituximab, n (%) | 2 (0.28) | 2 (0.31) | 0 (0) | 1.000 |
| Anticoagulation therapy |  |  |  |  |
| Anticoagulants medicine |  |  |  |  |
| Currently on warfarin, n (%) | 38 (5.40) | 19 (3.03) | 19 (24.6) | < 0.001 |
| Currently on direct oral anticoagulant, n (%) | 10 (1.42) | 9 (1.43) | 1 (1.29) | 1.000 |
| Antiplatelets medicine |  |  |  |  |
| Currently on single antiplatelet therapy, n (%) | 115 (16.3) | 72 (11.4) | 43 (55.8) | < 0.001 |
| Currently on dual antiplatelet therapy, n (%) | 8 (1.14) | 2 (0.31) | 6 (7.79) |  |
| Currently on triple antiplatelet therapy, n (%) | 1 (0.14) | 0 (0) | 1 (1.29) |  |

Abbreviations: *aPL*, antiphospholipid antibody; *APS*, Antiphospholipid antibody syndrome; *aCL*, anticardiolipin antibody; *aβ2GPⅠ*, anti-β2GPⅠ antibody; *AZA,* Azathioprine; *csDMARDs*, conventional synthetic disease-modifying anti-rheumatic drugs; *CNS*, Central Nervous System; *HCQ*, Hydroxychloroquine; *IGU*, Iguratimod; *IL-6R*, Interleukin-6 receptor; *IS*, immunosuppressant; *IVCY*, Intravenous cyclophosphamide; *IVIG*, Intravenous immunoglobulin; *LA*, Lupus anticoagulant; *LEF*, Leflunomide; *MMF*, Mycophenolate mofetil; *mPSL*, Methylprednisolone; *MTX*, Methotrexate; *MZR*, Mizoribine; *POCY*, per oral cyclophosphamide; *SASP*, Salazosulfapyridine; *TAC*, Tacrolimus; TNFα, Tumor necrosis factor α; *JAK*, Janus kinase.

*^1^* n (%) for categorical data; mean ± SD or median (IQR) for qualitative data.

*^2^* Pearson chi-square test (or chi-square test with the Yates continuity, or Fisher exact test if appropriate).

^a^ Digital vascular complications involve finger thrombosis or gangrenes, excluding digital ulceration related to scleroderma diagnosed by clinicians.

^b^ Abdominal arterial thrombosis included thrombosis involving the abdominal aorta and the branch.

^c^ Venous thrombus embolism had lower limbs venous thrombus embolism or pulmonary embolism.

^d^ Premature birth before the 34^th^ week of gestation due to hypertensive disorders of pregnancy with preeclampsia or placental insufficiency.
